# Comparison of two high-throughput semiconductor chip sequencing platforms in noninvasive prenatal testing for Down syndrome in early pregnancy

**DOI:** 10.1186/s12920-016-0182-9

**Published:** 2016-04-30

**Authors:** Sunshin Kim, HeeJung Jung, Sung Hee Han, SeungJae Lee, JeongSub Kwon, Min Gyun Kim, Hyungsik Chu, Hongliang Chen, Kyudong Han, Hwanjong Kwak, Sunghoon Park, Hee Jae Joo, Byung Chul Kim, Jong Bhak

**Affiliations:** GenomeCare, Suwon, Republic of Korea; Mirae & Heemang OB/GYN Clinic, Seoul, Republic of Korea; Seoul Clinical Laboratories (SCL), Yongin, Republic of Korea; Namujungwon Maternity Hospital, Yangju, Republic of Korea; GN Maternity Hospital, Pyeongtak, Republic of Korea; Xiamen Vangenes BioTech, Xiamen, Fujian China; Department of Nanobiomedical Science, BK21 PLUS NBM Global Research Center for Regenerative Medicine, Dankook University, Cheonan, Republic of Korea; TheragenEtex, Suwon, Republic of Korea; The Genomics Institute (TGI), BioMedical Engineering, UNIST, Ulsan, 687-798 Republic of Korea; Geromics, Ulsan, 687-798 Republic of Korea; Genome Research Foundation, Osong, Chungbuk Republic of Korea

**Keywords:** Non-invasive prenatal testing, Sequencing, Circulating fetal DNA, Trisomy, Genome

## Abstract

**Background:**

Noninvasive prenatal testing (NIPT) to detect fetal aneuploidy using next-generation sequencing on ion semiconductor platforms has become common. There are several sequencers that can generate sufficient DNA reads for NIPT. However, the approval criteria vary among platforms and countries. This can delay the introduction of such devices and systems to clinics. A comparison of the sensitivity and specificity of two different platforms using the same sequencing chemistry could be useful in NIPT for fetal chromosomal aneuploidies. This would improve healthcare authorities’ confidence in decision-making on sequencing-based tests.

**Methods:**

One hundred and one pregnant women who were predicted at high risk of fetal defects using conventional prenatal screening tests, and who underwent definitive diagnosis by full karyotyping, were enrolled from three hospitals in Korea. Most of the pregnant women (69.79 %) received NIPT during weeks 11–13 of gestation and 30.21 % during weeks 14–18. We used Ion Torrent PGM and Proton semi-conductor-based sequencers with 0.3× sequencing coverage depth. The average total reads of 101 samples were approximately 4.5 and 7.6 M for PGM and Proton, respectively. A Burrows-Wheeler Aligner (BWA) algorithm was used for the alignment, and a z-score was used to decide fetal trisomy 21. Interactive dot diagrams from the sequencing data showed minimal z-score values of 2.07 and 2.10 to discriminate negative versus positive cases of fetal trisomy 21 for the two different sequencing systems.

**Results:**

Our z-score-based discrimination method resulted in 100 % positive and negative prediction values for both ion semiconductor PGM and Proton sequencers, regardless of their sequencing chip and chemistry differences. Both platforms performed well at an early stage (11–13 weeks of gestation) compared with previous studies.

**Conclusions:**

These results suggested that, using two different sequencers, NIPT to detect fetal trisomy 21 in early pregnancy is accurate and platform-independent. The data suggested that the amount of sequencing and the application of common, simple, and robust statistical analyses are more important than sequencing chemistry and platform types. This result has practical implications in countries where PGM is approved for NIPT but the Proton system is not.

## Background

Recently, early stage prenatal screening to detect fetal aneuploidy has become common for pregnant women [1, 2]. Women over the age of 35 have an increased risk of giving birth to an abnormal baby; hence, accurate prediction-based tests for fetuses are required. Several prenatal screening methods are in use. Common first-trimester screening comprises a combination of ultrasound and maternal serum markers [3]. Women at high risk for fetal chromosome abnormalities have the option to undergo invasive prenatal diagnostic tests such as chorionic villus sampling (CVS) at 10–13 weeks gestational age or amniocentesis at 15–18 weeks gestational age [4, 5]. However, these tests are reported to be associated with iatrogenic pregnancy loss [6].

Decades ago, researchers discovered that cancer DNA could be detected as both circulating tumor cells and cell-free DNA (cfDNA) in human blood. Using this observation, researchers proposed that fetal DNA could be detected using molecular assays and cell-free fetal DNA (cffDNA) was indeed detected in maternal blood [7]. Today, women can choose noninvasive prenatal testing (NIPT) of cffDNA [8]. CffDNA is generally produced from apoptotic trophoblasts in the placenta [7] during pregnancy, and the amount of cffDNA increases with gestational age. During weeks 10–22 of gestation, approximately 10 % of free DNA in the mother’s plasma is estimated to be cffDNA. There is a very high level of variance in the fraction and amount of cffDNA among individuals [9]. Therefore, applying molecular testing requires an extremely accurate detection approach or a massive amount of data to overcome this detection difficulty. Our previous work [10] showed that it is possible to detect fetal chromosome abnormalities for pregnant women in weeks 12–21 of gestation by amplifying and producing a large number of DNA fragments for quantitative analyses. The percentage of cffDNA is proportional to gestational age; therefore, it is important to perform the test at the right time. At the early stage of pregnancy, the fraction of fetal DNA is perhaps the most important factor for NIPT, because the common aneuploidies are very difficult to detect for pregnant women in the early weeks of gestation [11] because of the lack of fetal DNA.

To carry out efficient NIPT, it is necessary to understand the limitations and characteristics of cffDNA. For example, cffDNA is normally only around 150 base pairs (bp) [12]. Also, cffDNA has a short half-life of about 16 min [13, 14].

NIPT technologies have been well accepted because of two critical clinical benefits: there is no risk of pregnancy loss and NIPT can be used as an early pregnancy test compared with amniocentesis; however, discordant NIPT data resulting from placental or maternal cell mosaicism requires full karyotyping, which is the gold standard for aneuploidy tests, to confirm positive outputs [15–17].

Nowadays, next-generation sequencing (NGS) is widely used for NIPT [18–24]. Ion Torrent PGM from Life Technologies, a semiconductor-based sequencing platform assessed here for NIPT and compared with its later and larger-capacity version of Ion Torrent Proton, enables a reduced turnaround time of sequencing data to within 2 to 4 h for a clinical sequencing service. A recent study described the outcomes for noninvasive detection of common fetal trisomy 13, 18, and 21 using the ion semiconductor platform, Ion Proton, which produced greater than 98 % sensitivity and specificity [23]. However, there are many different types and versions of sequencers, making it necessary for granting authorities to evaluate the generality and robustness of such sequencers for NIPT.

As far as we know, there has been no direct comparison between PGM and Proton sequencers for NIPT with large sample sizes. Another reason to compare PGM and Proton sequencers is that PGM is faster. The turnaround time for clinical applications is very important, and ion semiconductor sequencers, such as PGM, have been widely accepted because of their simplicity and speed. Prenatal testing using PGM has become feasible for the noninvasive detection of fetal aneuploidy [25]. PGM machines are small and inexpensive; therefore, comparing the benefits and overall accuracy of both PGM and Proton in terms of data produced could be important clinically for doctors who would like to use NIPT. The experimental cost of the bench-top PGM sequencer is much lower than other common sequencers, although PGM’s general sequencing accuracy is lower. From Ion 314/316/318 chips of PGM, 70 Mbp to 2 Gbp of raw sequencing data can be produced, and 85 % of the reads usually reaches Q20, a common sequencing quality threshold. With the Ion Proton platform, nearly 10 Gbp of raw sequencing data are produced with approximately 75 % of them reaching Q20. This amount is sufficient to determine the quantitative variation caused by chromosomal abnormality. A recent study confirmed that high-throughput ion semiconductor sequencing was feasible in noninvasive prenatal testing of fetal aneuploidies [10, 23]. In that study, only eight maternal plasma DNA samples were used, comprising four normal pregnancies and four with trisomy 21 fetuses, which were sequenced on Ion Torrent 314/316/318/PI chips [26]. Separately, using different samples, Ion Proton sequencer-based results have also demonstrated successfully that ion semiconductor sequencing is suitable for NIPT [10, 23].

Here, we compared the Ion Torrent PGM and Proton platforms for NIPT for fetal trisomy 21 directly using PGM and Proton simultaneously for the same set of samples to provide some general applicability of ion semiconductor-based sequencers. We were also interested in demonstrating whether any sequencers could discriminate fetal aneuploidy from normal chromosomes in blood, even if the fetal DNA fraction is not sufficient for NIPT. In doing so, we tried to detect fetal trisomy 21 for the pregnant women, most of whom (69.79 %) were at weeks 11 to 13 of gestation.

## Methods

### Study subjects

From December 2014 to April 2015, 101 pregnant women aged between 25 and 42 years (Table 1) were enrolled under an Institutional Review Board protocol in three hospitals (Mirae & Heemang, Namujungwon, and GN in Korea) after high-risk group screening. Sixty-seven of them (69.79 %) were at weeks 11–13 of gestation, and 29 (30.21 %) were at weeks 14–18 of gestation. Participants underwent invasive diagnostic testing (amniocentesis) for fetal karyotyping, the results of which were blinded. Before amniocentesis, they agreed to participate in this study, donated their blood samples, and provided written informed consent.

**Table 1:** Demographic characteristics. Demographic characteristics of 101 pregnant women in Mirae & Heemang, Namujungwon, and GN hospitals in Korea

Demographic characteristics	Euploid (*n* = 96)	T21 (*n* = 5)	*P* value	Total (*n* = 101)
Maternal age, years, mean ± SD	35.55 ± 3.63	33.40 ± 3.64		35.45 ± 3.64
≥35 years (%)	59 (61.46)	1(20.00)		60 (59.41)
NIPT during gestational week 11–13 (%)	67 (69.79)	3 (60.0)		70 (69.31)
NIPT during gestational week 14–18 (%)	29 (30.21)	2 (40.0)		31 (30.69)
PGM, z-score of chr21 (min, max)	−3.46, 2.07	5.50, 9.43	<0.0001†	−3.46, 9.43
Proton, z-score of chr21 (min, max)	−2.32, 2.10	6.20, 8.86	<0.0001†	−2.32, 8.96

*T21* Trisomy 21, *SD* standard deviation, *NIPT* non-invasive prenatal testing. *P* values from †Student’s *t*-test

We used the outcomes of the standard prenatal aneuploidy screening with individual risk scores and interpretations generated by accredited clinical laboratories to identify the group at high risk of fetal defects. First-trimester serum markers included pregnancy-associated plasma protein A (PAPP-A) and free beta subunit or total human chorionic gonadotropin (hCG). A low level of PAPP-A and high hCG might indicate Down syndrome. First-trimester serum markers were used in combination with sonographic measurement of fetal nuchal translucency to classify the women into high- or low-risk groups. The second-trimester serum test, termed quadruple screening, was used alone to evaluate and define aneuploidy risk. Chromosomal abnormality was tested with cultured fetal cells from amniotic fluid, as described by Barch et al. [27]. The result of cytogenetic analysis on all 101 pregnant women indicated that 96 (95.0 %) were chromosomally normal, and five (5.0 %) had trisomy 21.

### CfDNA preparation and massively parallel short read sequencing

More than 10 mL of peripheral blood was collected and stored in a BCT™ tube (Streck, Omaha, NE, USA). The blood sample was centrifuged at 1200 × g for 15 min at 4 °C. The plasma portion was transferred to microcentrifuge tubes and centrifuged again at 16,000 × g for 10 min at 4 °C. One mL of plasma was used to extract cfDNA, using a QIAamp Circulating Nucleic Acid Kit (Qiagen, Netherland). The end-repair of the plasma cfDNA was performed using T4 DNA polymerase, Klenow DNA polymerase and T4 polymerase kinase. DNA libraries for the Ion PGM and Proton sequencing systems were prepared according to the protocol provided by the manufacturer (Life Technologies, SD, USA). PGM 318 and Proton PI Chip Kit version 2.0 were used to produce an average of 0.3× sequencing coverage depth per nucleotide. Barcode Indexing was used in both PGM and Proton chips. The index served as a token to differentiate each sample from the multiplexed sample mixtures.

### Data analysis

DNA fragments with different lengths derived from the Ion Torrent Suite software were trimmed from the 3' end using a sequencing quality value of >15 and filtered by read length (<50 bp) and GC contents (35–45 %). The Picard program (http://broadinstitute.github.io/picard/) was used to remove duplicate DNA reads. The sequence fragments from each sample were then mapped to the human reference genome sequence (hg19). We evaluated BWA [28], Bowtie [29], and SOAP2 [30] mapping software and chose BWA to acquire the final mapping results. Every chromosome was divided into segments with a bin size of 300 kb to calculate the z-scores to determine trisomy 21. For all 101 samples, we calculated the z-score for each chromosome of each sample to detect the aneuploidy with mapped reads, as well as the average mapped reads and standard deviation (SD) of 96 euploid samples. The z-score of case 1 for chr21, for example, was calculated as follows: z-score_chr21_case1_ = (mapped reads of chr21_case1_ – mean mapped reads of chr21_euploid group_)/(SD for mean mapped reads of chr21_euploid group_). The minimal z-scores to determine negative versus positive cases of trisomy were > 2.07 and > 2.10 for fetal trisomy 21 for PGM and Proton systems, respectively. We used Student’s *t*-test to evaluate the statistical significance of the comparison between the euploid and T21 groups, and a value < 0.05 was considered statistically significant.

## Results

**Fig. 1:**
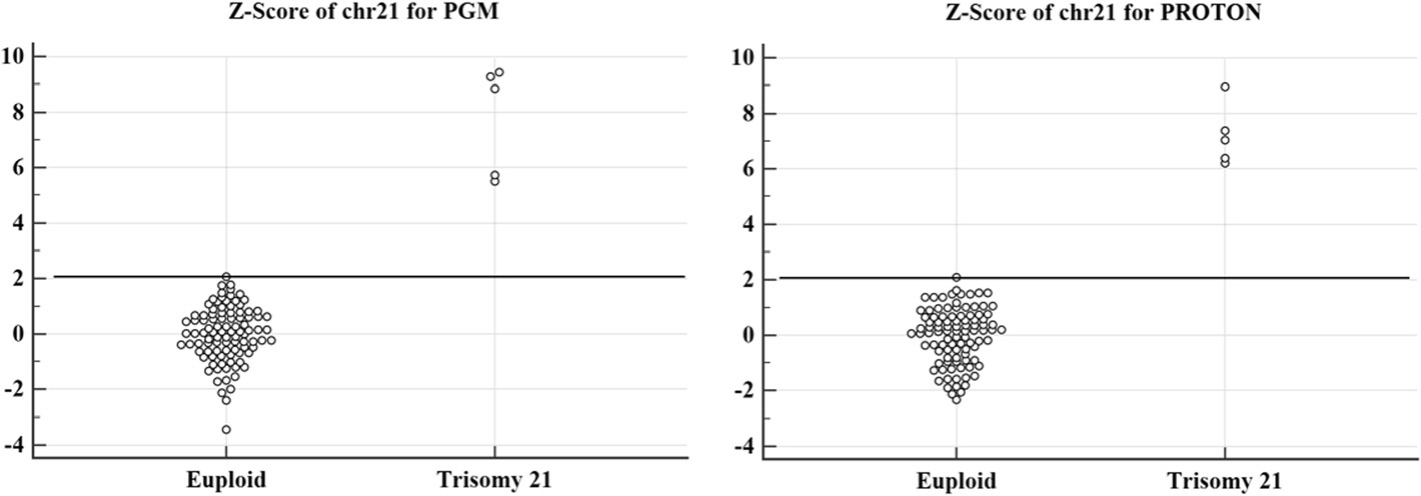
Dot diagrams. Interactive dot diagrams of trisomy 21 for PGM and Proton sequencers showing the minimal z-scores

**Fig. 2:**
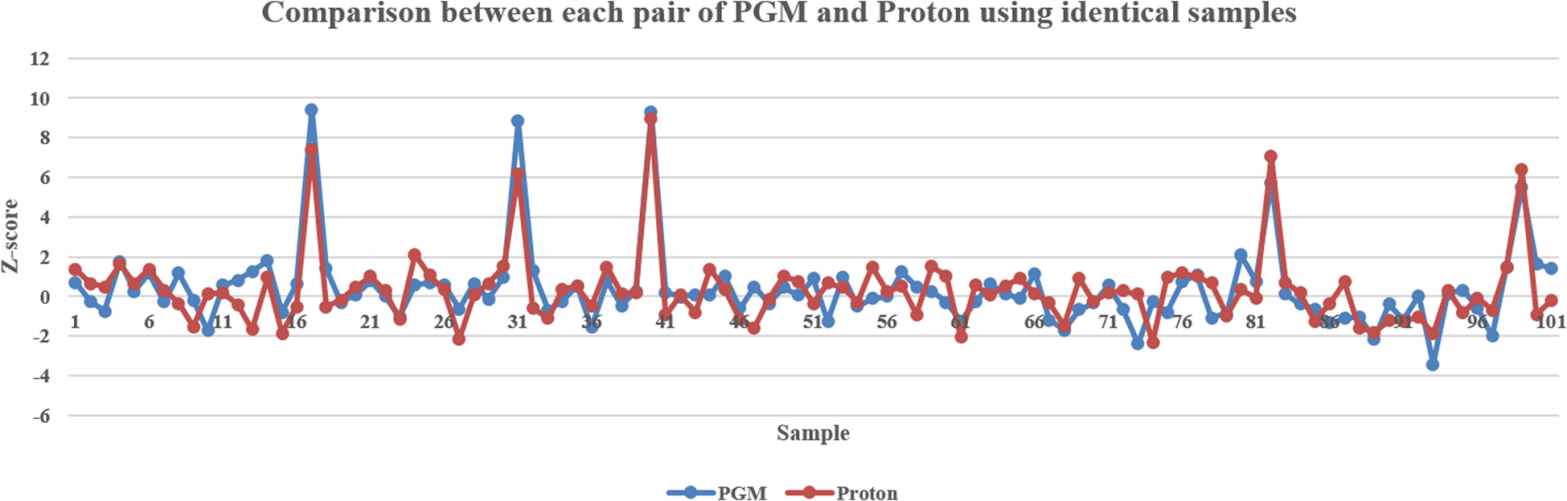
Z-score comparison between PGM and Proton platforms using identical samples

By comparing the sequencing results, we found that the sequencing qualities of both platforms were slightly different, although they are very similar sequencers from the same company. The average total reads of the 101 samples were approximately 4.5 and 7.6 M for PGM and Proton, respectively. For the 318 chip of the PGM sequencer, the average read mapped ratio was higher with a lower SD, the mean read length was longer with a higher SD and the Phred quality score was higher with a higher SD. The Proton PI chip had a better correlation coefficient between the chromosome length and the total reads of the corresponding chromosome. However, we observed that the sequencing quality differences between both platforms did not affect the final z-score results, indicating that the number of DNA reads is more important than individual sequence fragment quality. Interactive dot diagrams for fetal trisomy 21 for the PGM and Proton systems showed the smallest z-score values of 100 % positive predictive value (PPV) and negative predictive value (NPV) (Fig. 1). Figure 2 shows a comparison for an identical sample sequenced by PGM and Proton platforms. The z-scores of the negative samples showed almost the same trends between the platforms, while those of the positive samples showed the same trends. For PGM, the smallest z-score value of 2.07 showed a 100 % PPV and NPV. The minimal z-score of 2.10 was used to classify negative versus positive cases for the Proton system. Table 2 shows the PPVs and NPVs of the NIPT outcomes for fetal trisomy 21 for PGM and Proton, respectively. For both PGM and Proton, the PPV (95 % CI: 47.8–100.0 %) and NPV (95 % CI: 96.2–100.0 %) were 100 %.

**Table 2:** Positive and negative predictive values. The positive and negative predictive values of the NIPT results for fetal trisomy 21 for the PGM and Proton sequencers used in this study

Chip	Positive predictive value (95 % CI)	Negative predictive value (95 % CI)
318 of PGM	100.0 % (47.8–100.0 %)	100.0 % (96.2–100.0 %)
PI of Proton	100.0 % (47.8–100.0 %)	100.0 % (96.2–100.0 %)

## Discussion

We compared the PGM with Proton ion semiconductor systems by performing NIPT using cfDNA in a high-risk population. Comparison of the PGM 318 and Proton PI chips was performed with five different parameters to measure their sequencing quality, which revealed sequencer-specific differences (Table 3). However, the two different platforms showed the same accuracy (100 %) using the same set of samples. Our results suggested that prenatal prediction of Down syndrome could be performed equally well by both semiconductor-based sequencing platforms. In addition, we found that early fetal aneuploidy detection (weeks 11–13 of pregnancy) is possible, in addition to detection in the late stage of pregnancy.

**Table 3:** Comparison of both Ion chips. Comparison of PGM and Proton semiconductor-based Ion chips using five different factors

Chip type	Total reads		Read mapped ratio		Mean read length		Phred quality score (≥Q20)		Correlation	
	Average	SD/average	Average	SD	Average	SD	Average	SD	Coefficient	Significance
318 of PGM	4512893	0.2823	99.078	0.6635	147.871	10.693	0.8611	0.0374	0.9704	*P* < 0.0001
PI of Proton	7572314	0.1837	99.011	1.1470	147.842	5.669	0.8537	0.0225	0.9813	*P* < 0.0001

*SD* standard deviation

Previous studies detected no quality differences between Ion Torrent sequencer and the nucleotide synthesis-based Illumina sequencers by comparing PGM data vs*.* HiSeq2000- and MiSeq-derived data, although the error rate of PGM data was relatively higher than those of the HiSeq2000 and MiSeq data [31, 32]. Chen et al. [33] evaluated the performances of Proton and MiSeq systems for extremely low-coverage sequencing, and showed that both sequencers detected aneuploidies correctly. Wang et al. concluded that the data quality of Ion Torrent PGM was generally better than that of the Proton system [26]. However, they showed that both PGM- and Proton-based semiconductor high-throughput sequencing was feasible in the noninvasive prenatal testing of fetal aneuploidies [26], although it was carried out with a very small number of samples. In our previous study, we showed that the detection of fetal T18 and T21 could be carried out using the Ion Torrent Proton system [10]. A large-scale clinical study by Liao et al. also showed that NIPT using an Ion Proton sequencer could be successful [23]. In addition, the Ion Proton system can generate about 80 million raw reads in 3–4 h, allowing chromosomal aneuploidy detection in 2 working days. Hence, it could be suitable for operations that require fast and accurate turnaround times [33].

Conventional prenatal screening is not perfect and its detection accuracy for chromosomal aneuploidy is below 100 %. Screens using maternal serum markers and ultrasound have been approved, but have lower accuracy. Henry et al. [34] analyzed Down syndrome births after routine noninvasive screening, based on conventional, non-sequencing tests, which have mostly replaced age-related invasive processes. They found that despite the increase in prenatal screening, newborn children with Down syndrome increased in women over the age of 35 years, because the mothers believed that a single blood test would be sufficient to detect trisomy 21.

A positive NIPT result should include a follow-up test with an invasive prenatal method to confirm the fetal chromosomal aneuploidies. Accurate detection of common chromosomal aneuploidies, particularly T18 and T21, has been consistently reported in previous works of NIPT using cfDNA [18–23]. The current price of a plasma cfDNA test is $800–2000 in the US and $500–1500 in some other countries [8]. Two previous studies evaluated the cost of a cfDNA test in women with positive prevalent screening outcomes and concluded that the use of the cfDNA test was associated with a net price reduction compared with conventional CVS or amniocentesis [35, 36]. One limitation of our current study is that it could not detect additional positive cases for other common chromosomal aneuploidies, such as T18 and T13.

In terms of the time to perform NIPT, a previous study [10] reported that for high sensitivity and specificity, the samples should be collected during weeks 14–21 of gestation. It is advantageous to find a chromosomal abnormality at an early stage of pregnancy; therefore, it is crucial to investigate whether a similar accuracy could be achieved in early pregnancy to expand the clinical utility of NIPT. Our current results indicated that both semiconductor-based platforms are sufficiently sensitive and effective for pregnant women, most of which were at 11 to 13 weeks of gestation.

Although both the PGM and Proton platforms yielded similar results for NIPT, using the minimal z-scores thresholds identified here for classification cannot be a standard measure, because there is usually a grey zone where the classification is not certain and further investigation is required. Therefore, a larger sample size is required to confirm the reliability and validity of our results.

## Conclusions

We showed that fetal trisomy 21 could be detected successfully by two different ion semiconductor sequencers (Ion Torrent PGM and Proton) and confirmed that both Ion chips are suitable for cfDNA screening for pregnant women at an early stage. In Korea, PGM is approved as a medical instrument for cfDNA testing, while the Proton awaits approval. Therefore, our investigation provided evidence of PGM’s applicability to public clinical tests. As PGM has been used for NIPT for some years, these results could be used in other countries where NIPT is provided to pregnant women.

### Availability of data and materials

The information supporting the conclusions of this article is included within the article.

## Abbreviations

Bp: base pairs
cfDNA: cell-free DNA
cffDNA: cell-free fetal DNA
CVS: chrorionic villus sampling
hCG: human chorionic gonadotropin
NGS: next-generation sequencing
NIPT: non-invasive prenatal testing
NPV: negative predictive value
PPV: positive predictive value
SD: standard deviation
